# Correlation between COVID-19 and its manifestations in the oral cavity

**DOI:** 10.6026/973206300211750

**Published:** 2025-06-30

**Authors:** Bhushan Sharma, Jagatishi Kaur, Rajveer Mann, George Koshy, Sonal Grover, Deepti Sharma

**Affiliations:** 1 Department of Oral and Maxillofacial Pathology, Christian Dental College & Hospital, Ludhiana, Punjab, India

**Keywords:** COVID-19, oral cavity, oral manifestations, taste disorders

## Abstract

The prevalence of oral manifestations among COVID-19 patients is of interest. This is a cross-sectional, questionnaire-based study conducted among 23 recovered COVID-19 patients aged over 18 years. The results showed the prevalence of oral manifestations up to 45%, with ageusia as the major outcome in 60% of the positive cases. These manifestations were predominantly observed in moderate to severe cases of COVID-19 among male patients above 50 years of age. More studies with sufficient sample sizes and broader regional representation are recommended to validate these observations.

## Background:

Coronavirus disease 2019 (COVID-19) is a viral infection that progresses from the upper to the lower respiratory tract, transmitted through human-to-human contact. The causative agent of the disease was identified as severe acute respiratory syndrome coronavirus 2 (SARS-CoV-2), a novel human-infecting beta coronavirus, which initiated the pandemic that commenced in Wuhan, China, and swiftly disseminated globally, with India being one of the most severely impacted countries [1, 2]. SARS-CoV-2 can enter through the oral mucosa via salivary gland receptors, possibly causing early symptoms like loss of smell, taste, and dry mouth before typical COVID-19 signs appear [3]. Initially, it was posited that the absence of oral involvement constitutes a distinguishing characteristic of COVID-19 exanthema compared to other viral exanthemas. Recent studies suggest that the coronavirus infiltrates human cells through the angiotensin-converting enzyme 2 (ACE2) receptors by creating a risk map of various organs utilizing single-cell RNA sequencing (scRNA-seq) datasets [4]. Consequently, cells exhibiting ACE2 receptor distribution may serve as host cells for the virus, resulting in an inflammatory response in associated organs and tissues, including the tongue mucosa and salivary glands [5, 6]. Various lesions have been documented in the oral cavity associated with COVID-19, including taste disturbances, ulcers, desquamative gingivitis, petechiae and concomitant conditions such as candidiasis [7]. Further findings encompass periodontitis, mucormycosis, fissured or depapillated tongue, halitosis, abnormal pigmentation, oedema and spontaneous oral hemorrhage. The most frequently impacted areas are the tongue, palate, lips, gingiva and buccal mucosa, with the tongue being the most affected, followed by the labial mucosa and palate [8, 9]. Since the prevalence of clinical manifestations of the disease is still unknown, the range of its manifestations in the oral cavity has been considered of immense interest. Therefore, it is of interest to evaluate the prevalence of oral manifestations in patients diagnosed with COVID-19.

## Materials and Methods:

## Study design:

A cross-sectional, questionnaire-based study was carried out among COVID-19 recovered patients regarding health status and symptoms in oral cavity before, during and after the disease illness in the In-Patient Department (IPD) of Medical Hospital and outpatient department (OPD) of Dental hospital in North India to find the prevalence of oral manifestation and to describe the pattern of the same. Anonymity and confidentiality of the patients were maintained.

## Inclusion criteria:

[1] Participants with a confirmed history of COVID-19 infection, who have recovered within a period of 2 weeks to 6 months prior to enrolment in this study.

[2] Patients above 18 years of age.

[3] Patients who agreed to participation and have provided written consent.

## Exclusion criteria:

[1] Patients having any common symptoms related to COVID-19 like fever, cough, cold, *etc.*, at the time of investigation.

[2] Patients having lesions associated with habits or drugs.

[3] Patients with history of chronic systemic disease/any debilitating disease.

## Sample size:

A convenient sample of 23 subjects, who were previously admitted in the hospital and OPD of CDC were selected on the basis of inclusion and exclusion criteria. Data was collected between 2 weeks to 6 months from the date of diagnosis of COVID-19 disease, which was confirmed by positive RT-PCR reports provided by the subjects.

## Data collection:

Data for the study was collected from the participants through personal visits or online via a pdf questionnaire. Majority of subjects provided the required clinical history directly to the examiner in dental OPD. All the necessary preventive measures (*e.g.*; use of personal protective equipment (PPE), face masks, strict hand hygiene) were strictly adhered to in order to reduce the risk of disease transmission.

## Survey tool:

Questionnaire: A validated, close-ended questionnaire consisting of 12 questions was employed to collect data on the health status and symptoms of the buccal cavity prior to, during, and following the disease manifestation.

## The following information was gathered from study subjects:

[1] Demographic Details: Name, Age, Gender and address of the subject were gathered.

[2] Disease-related: date of diagnosis, history of hospitalization and vaccination status of COVID.

[3] Health and habit history: the subject was asked regarding the presence of any other debilitating disease (*e.g.*, hypertension, diabetes) and a history of personal habits.

[4] Oral manifestations like xerostomia, halitosis, burning sensation and painful ulcerations, its time of onset and duration.

[5] Any history of dental check-up before, during or post the infection.

## Data synthesis:

Data was collected in the form of questionnaires and subsequently compared with was with the oral findings to determine the presence or absence of oral manifestations associated with COVID-19. The primary outcome of interest was the prevalence of these oral manifestations. Oral lesions in patients with COVID-19 were documented and categorized broadly based on their anatomical location. Quantitative analysis was performed to evaluate the findings.

## Results:

Of the 23 subjects examined, 10 resulted positive for oral manifestations related to COVID-19. The various manifestations included ageusia, halitosis and depapillated tongue, Pigmentation of the buccal and labial mucosa, desquamative gingivitis, fissured tongue and irregular ulcer. The most prevalent finding was ageusia which was found among 7 out of the 10 subjects who were positive for the findings (Table 1 - see PDF). Out of the 10 patients, 6 patients (60%) were aged above 50 years of age with a male to female ratio of 7:3, suggesting a higher prevalence in male population (Table 2 - see PDF). Six patients were hospitalized during the infection. Three patients were on antidiabetic medication and two patients were on antihypertensive medication. The common sites involved for oral manifestations were tongue, palate, buccal mucosa, labial mucosa and gingiva (Table 3 - see PDF).

## Discussion:

The most prevalent oral symptoms in COVID-19 patients were taste disorders, which is consistent with the literature. Nicola Cirillo *et al.* (2021) [10] conducted a study that provided compelling evidence that gustatory changes are cardinal symptoms of COVID-19. Binmadi *et al.* (2022) [11] identified distortion of taste among 60% of patients, which is also in agreement with the results established in this study. Taste disorders are further classified into three categories, based on quantitative measures, which includes hypogeusia, ageusia and dysgeusia. This particular discovery was of interest to COVID-19 due to the fact that these conditions were not reported during previous SARS outbreaks. One symptom that has been repeatedly reported in COVID-19 patients across Europe and Asia is the loss of scent (anosmia) and taste (ageusia), as equally hypothesised by Rusell *et al.* (2020) [12]. The majority of the patients in this study presented with Ageusia. Hopkins *et al.* (2020) [13] identified anosmia and ageusia as viral manifestations in approximately 40% of cases. Although our study did not specifically assess olfactory disturbances, ageusia was reported in a notably higher proportion of subjects (70%). Consequently, taste disorders, which are easily detectable and early in onset, have the potential to serve as a useful indicator for patient screening and to facilitate self-isolation, thereby directly contributing to the rapid transmission of the disease. Carreras-Presas *et al.* (2020) [14] described diverse forms of oral mucosal lesions (ulcer, vesicle, bulla and desquamative gingivitis) that are clinically comparable to those observed in other viral mucocutaneous infections, such as herpes simplex, herpes zoster, or immunological disorders. In a 2020 systematic review encompassing 18 studies, Cuevas-Gonzalez *et al.* reported that dysgeusia, xerostomia, and burning mouth sensation were the most frequent early manifestations following SARS-CoV-2 infection while the predominant clinical findings included ulcerative lesions, persistent dysgeusia, and opportunistic infections such as Candida albicans [15]. The potential overlap in clinical presentation was further supported by the identification of similar oral lesions in the present study. This study found that the majority of positive cases were among elderly adults, representing over 50% of the total. Iranmanesh *et al.* (2020) [8] confirmed that a greater number of older individuals exhibited oral symptoms. Age-related immune suppression may account for the increased occurrence of oral symptoms in this demographic. Paradowska-Stolarz *et al.* (2021) [9] noted that older patients and those with a greater severity of illness experienced more severe episodes of oral lesions. The increased occurrence of oral symptoms in female participants may be ascribed to hormonal regulation, given that sex hormones are recognised to affect immune responses and mucosal health, as indicated by Taneja (2018) [16]. Another conclusion was that oral signs occurred irrespective of the severity of COVID-19. A research by Ganesan *et al.* (2022) [17] revealed that most oral symptoms were associated with severe COVID-19. This study does not provide evidence in support of the proposed hypothesis. Studies also recorded fungal infections in many extremely severe COVID-19 cases [18]. No prevalence of any fungal infection was observed in this study. Nevertheless, this study, like others on the subject, has certain limitations. One significant concern is the potential for some oral manifestations to have gone unnoticed by patients during the course of infection, and consequently, not being captured in the recorded history or data. Additionally, the absence of detailed information regarding the duration and frequency of self-reported symptoms limited the ability to analyse oral manifestations comprehensively. The sample size was relatively small with a limited number of subjects, combined with variability in the severity of COVID-19 among participants, contributed to data heterogeneity and increased the risk of bias. A study conducted by Alhamed *et al.* (2023) [19] observed that most patients developed oral mucosal injuries during the hospitalization period, a finding that is also supported by the present study. This corroborates the hypothesis that such lesions may result from coinfections, impaired immunity, or adverse reactions to medications used in the treatment of COVID-19. These findings underscore the importance of integrating systematic oral evaluations into the standard care protocols for patients with infectious diseases. As highlighted by Charitos *et al.* (2020) [20], comprehensive clinical assessments are crucial in managing COVID-19, emphasizing the need for thorough evaluations of all potential manifestations, including those in the oral cavity. Oral lesions have been infrequently described in clinical studies on COVID-19. Among the published reports, taste alterations appear to be the most common oral manifestation, followed by ulcers, fissured tongue and desquamative gingivitis. The worldwide incidence of gustatory impairment among COVID-19 patients is believed to be around 45%. Nevertheless, there remains a deficiency of evidence about oral mucosal lesions in individuals with COVID-19 and their corresponding etiopathogenesis.

## Conclusion:

Routine oral examination in suspected or recovered patients is critical. Symptoms like ageusia, though often overlooked, can aid in timely management, isolation measures and help in understanding of the full clinical spectrum of the disease. The predominance of these manifestations in older adults and males suggests a possible link with age-related immune decline and gender-based immunological differences. Further studies with sufficient sample sizes and broader regional representation are recommended to validate these observations.
